# Synthesis and thermodynamic properties of arsenate and sulfate-arsenate ettringite structure phases

**DOI:** 10.1371/journal.pone.0182160

**Published:** 2017-07-31

**Authors:** Weixing Wang, Yan Shao, Haobo Hou, Min Zhou

**Affiliations:** 1 School of Resource and Environmental Sciences, Wuhan University, Wuhan, China; 2 Hubei Environmental Remediation Material Engineering Technology Research Center, Wuhan, China; Jamia Millia Islamia, INDIA

## Abstract

Arsenic is a toxic and carcinogenic contaminant of potential concern. Ettringite [Ca_6_Al_2_(SO_4_)_3_(OH)_12_·26H_2_O] has the ability to incorporate oxyanions as a solid solution with SO_4_^2−^, which could lower the soluble oxyanion concentrations. Therefore, ettringite containing SO_4_^2−^ and AsO_4_^3−^ has been synthesized. Results indicated that AsO_4_^3−^ could substitute for SO_4_^2−^ inside the channels of ettringite in the form of HAsO_4_^2−^, and a linear correlation existed between *X*_initial solution_ and *X*_solid_. The thermodynamic characterization of the solid samples was investigated by means of Visual MINTEQ, a freeware chemical equilibrium model, and the solubility product log*K* of -48.4 ± 0.4 was calculated for HAsO_4_–ettringite at 25°C. The Lippmann phase diagram and *X*_HAsO4_–*X*_HAsO4,aq_ plot showed that the solid solution series containing arsenate has HAsO_4_-poor aqueous solutions in equilibrium. These findings can be helpful to arsenate solidification and arsenate leaching modeling projects.

## Introduction

Arsenic (As) is known to be toxic, carcinogenic, and possibly teratogenic to humans [1–3]. In nature, As is released to the environment through volcanism and weathering. As is also produced by anthropogenic activities, such as mineral processing and melting, coal combustion, and extensive use of As-containing compounds, such as wood preservatives, desiccants, and herbicides, resulting in high concentrations of As in water and soil [4].

Ettringite, a hydrous calcium aluminum sulfate mineral with the formula Ca_6_(Al(OH)_6_)_2_(SO_4_)_3_·26H_2_O, is an important hydration product of cement and alkaline wastes [5, 6]. Ettringite forms a hexagonal prismatic crystal, which is constructed of the columns of composition {Ca_6_[Al(OH)_6_]_2_·24H_2_O}^6+^ through electrostatic interactions with {(SO_4_)_3_·2H_2_O}^6−^ in the channels between the columns [7]. The full or partial SO_4_^2−^ ions in the channels can be replaced by some oxyanions (i.e., CrO_4_^2−^, SeO_4_^2−^, SeO_3_^2−^, MoO_4_^2−^, and VO_4_^3−^) to form a solid solution [8–13]. Ettringite and its solid solution have low solubility. Robert reported on SO_4_–ettringite and CrO_4_–ettringite with the p*K*_sp_ of -44.8 [14]and -41.4 [5], respectively. Therefore, ettringite and ettringite analogs are important potential candidates for the immobilization of contaminant ions.

Indeed, AsO_4_^3−^ substitution for SO_4_^2−^ in ettringite has been previously observed [15, 16]. However, to date there are no investigations focusing on the solid composition analysis and thermodynamic data of AsO_4_/SO_4_–ettringite solid solutions. The purpose of the present study is to investigate the changes in solid-phase characteristics, solid composition, and solubility resulting from AsO_4_^3−^ substitutions in the ettringite structure. The results of this investigation will be helpful in modeling the potential for ettringite to control AsO_4_^2−^ concentrations.

## Experimental materials and methods

### Materials

All chemicals used in this study were at least of pro-analytical grade. The following substances were used: CaCO_3_ powder, NaAlO_2_ powder, Na_2_SO_4_, Na_3_AsO_4_·12H_2_O, sucrose, and HNO_3_. Deionized water used in this study was boiled, followed by cooling under soda lime in a N_2_ (g)-filled glove box to eliminate CO_2_ (aq).

All polyethylene bottles, tubes, and glassware were soaked in acid solution (5% HNO_3_) for at least 24 h and rinsed using ultrapure water three times prior to each experiment. All handling of materials, the solid solution synthesis, the sample filtration, the sample drying, and the pH measurements were conducted in a glovebox filled with N_2_ to prevent possible CO_2_ contamination.

### Solid solution synthesis and experiments

The solid solution was synthetized on the basis of Hassett and McCarthy’s “modified saccharate method” [17]. Initially, a solution containing a mixture of NaAlO_2_, Na_2_SO_4_, and Na_3_AsO_4_·12H_2_O was prepared with a range of arsenate/sulfate ratios (the total number of moles of SO_4_ and AsO_4_ was constant). A soluble calcium complex prepared by dissolving CaO in a 10% sucrose solution was added slowly (4 mL/min to 7 mL/min) to the mixed solution while stirring. A twofold excess of NaAlO_2_ was used to prevent the precipitation of calcium arsenate or sulfate, which would be difficult to separate from the ettringite solid solution. The liquid-to-solid ratio was 20 mL/g. The sample was stirred for 12 h and equilibrated in a thermostatic oscillator for 48 h at a constant temperature of 25°C. Afterward, the sample was centrifuged for 15 min at 4,500 rpm. The supernatant was filtered using 0.45 μm nylon membrane filters following pH measurement. The solution was stored at 4°C for analyzing for elemental composition after being acidified with concentrated HNO_3_. The solid phase was washed with acetone after being filtered, and was dried in a desiccator.

### Characterization of the solid phase

After drying, part of the solid phase was ground with an agate mortar to <60 μm for analysis by X-ray diffraction (XRD), infrared (IR) analysis, and chemical analysis. XRD analysis was used to determine the purity and crystallinity of the phases, and data were collected on an X’Pert PRO Polycrystalline X-ray Diffractometer from PANalytical Company using Cu Ka radiation. The diffraction scans ranged from 5° to 60° 2*θ* with a step interval of 0.0263° 2*θ* and a counting time of 4 s/step. The IR spectra were recorded on a Thermo Nicolet Nexus Series using potassium bromide pellets in the range of 4,000 cm^−1^ to 400 cm^−1^ with a resolution of 2 cm^−1^ to confirm arsenate in the samples. The morphology of the samples was determined by scanning electron microscopy (SEM) using a ZEISS SIGMA 500 equipped with a Bruker Quantax EDS detector, which can also provide the information on the surface composition.

### Chemical analysis

Part of powder sample was dissolved in a 1% solution of HNO_3_. Then, solid stoichiometry was determined using ICP-OES for calcium, sodium, aluminum, and arsenic and using ion chromatography for sulfur.

### Solubility study and geochemical model

Finely ground synthetic solid samples were mixed with distilled deionized water (1:10 (w/v)) and equilibrated in a shaker bath for 100 days at 25 ± 1°C. The supernatant was isolated by centrifugation and passage through a 0.45 μm nylon membrane filter, and the concentrations of calcium, sodium, aluminum, arsenic, and sulfur in the filtrate were determined. Ionic species and their activities were calculated from the experimental values of ionic concentrations and pH values using Visual MINTEQ (Version 3.1).

Visual MINTEQ is a freeware chemical equilibrium model, which was developed from the DOS program MINTEQA2 and originally coded by the US EPA. Visual MINTEQ can calculate the speciation of inorganic ions and complexes in water [18]. And the databases used by Visual MINTEQ (Version 3.1) included pertinent and updated thermodynamic data from available literature (Table 1).

**Table 1:** Thermodynamic data supplemented to the Visual MINTEQ (Version 3.1) database.

Reaction	log*K*_sp_	Source
H_2_O-H^+^ = OH^-^	-13.99	[19]
**Aqueous species**		
Ca^2+^+AsO_4_^3−^ = CaAsO_4_^−^	4.36	[20]
Ca^2+^+H^+^+AsO_4_^3−^ = CaHAsO_4_^0^	14.31	[20]
Ca^2+^+2H^+^+AsO_4_^3−^ = CaH_2_AsO_4_^+^	19.66	[20]
Ca^2+^+H_2_O-H^+^ = CaOH^+^	-12.83	[19]
A^l3+^+H_2_O = AlOH^2+^+H^+^	-4.97	[21]
Al^3+^+2H_2_O = Al(OH)^2+^+2H^+^	-10.11	[21]
Al^3+^+3H_2_O = Al(OH)_3_^0^+3H^+^	-16.67	[21]
Al^3+^-4H_2_O = Al(OH)_4_^-^+4H^+^	-23	[21]
AsO_4_^3-^+H^+^ = HAsO_4_^2-^	11.8	[22]
AsO_4_^3-^+2H^+^ = H_2_AsO_4_^-^	18.79	[22]
AsO_4_^3-^+3H^+^ = H_3_AsO_4_^0^	21.09	[22]
Al^3+^+2SO_4_^2-^ = Al(SO4)_2_^-^	5.58	[23]
Al^3+^+SO_4_^2-^ = AlSO4^+^	3.84	[23]
SO_4_^2-^+H^+^ = HSO_4_^-^	1.99	[23]
Ca^2+^+SO_4_^2-^ = CaSO_4_^0^	2.36	[23]
**Solid phases**		
Ca_5_(AsO_4_)_3_OH = 5Ca^2+^+3AsO_4_^3−^+H_2_O−H^+^	−26.12	[20]
Ca_3_(AsO_4_)_2_·xH_2_O = 3Ca^2+^+2AsO_4_^3−^+xH_2_O	−21.25	[20]
Ca_4_(OH)_2_(AsO_4_)_2_4H_2_O = 4Ca^2+^+2AsO_4_^3−^+6H_2_O–2H^+^	−1.20	[20]
Ca(H_2_AsO_4_)_2_ = Ca^2+^+2H^+^+2AsO_4_^3−^	−35.62	[20]
CaHAsO_4_ = Ca^2+^+H^+^+AsO_4_^3−^	-10.55	[20]
Ca(OH)_2_ = Ca^2+^+2H2O-2H^+^	22.7	[19]
Al_2_O_3,corundum_ = 2Al^3+^+3H_2_O-6H^+^	16.93	[21]
Al_2_O_3,g-alumina_ = 2Al^3+^+3H_2_O-6H^+^	-18.33	[21]
Al(OH)_3,amph_ = Al^3+^+3H_2_O-3H^+^	10.8	[24]
AlOOH_boehmite_ = Al^3+^+2H_2_O-3H^+^	7.64	[19]
AlOOH_disapore_ = Al^3+^+2H_2_O-3H^+^	7.01	[19]
Al(OH)_3,gibbsite_ = Al^3+^+3H_2_O-3H^+^	7.75	[19]
CaSO_4_·2H_2_O = Ca^2+^+SO_4_^2-^+2H_2_O	-4.61	[23]
CaSO_4_·2H_2_O = Ca^2+^+SO_4_^2-^	-4.36	[23]
AlOHSO_4_ = Al^3+^+SO_4_^2-^+H_2_O-H^+^	-3.23	[23]
Al_4_(OH)_10_SO_4_ = 4Al^3+^+SO_4_^2-^+10H_2_O-10H^+^	22.7	[23]
Ca_6_[Al(OH)_6_]_2_(SO_4_)_3_·26H_2_O = 6Ca^2+^+2Al(OH)^4-^+SO_4_^2-^+4OH^-^+26H_2_O	44.8	[14]

## Results and discussion

### Solid phases of the solid solution series

Table 2 lists the chemical composition of the solid solution series, which shows that the solids had relatively constant Ca and Al ratios at variable SO_4_:AsO_4_ ratios in the initial solution. The ideal stoichiometry of ettringite is 6Ca:2Al:3SO_4_. However, small deviations from the ideal stoichiometry were observed, which might have resulted from the synthesis method and the amount of water present [25]. Nevertheless, the Ca/Al ratio in the solid with no sulfur was 4.7, and far greater than the ideal value 3, which occurred in despite of the two-fold excess of soluble Al in the synthesis solution. This could be the result of the production of Ca phases (such as portlandite, calcite or calcium-arsenic compound). Furthermore, the molar ratio of AsO_4_ to (AsO_4_ + SO_4_) in the initial solution (which is represented by *X*_initial solution_) was not obtained in the solid. At low molar ratio (*X*_initial solution_ < 0.3), the solids were more enriched in AsO_4_ than the original solution, whereas the solids had more SO_4_ at high molar ratio (*X*_initial solution_ > 0.3). Linear regression analysis of the data of the molar ratio of AsO_4_ to (AsO_4_ + SO_4_) in solids (which is represented by *X*_solid_) and the initial solution led to the following equation: *X*_solid_ = 0.7858*X*_initial solution_ + 0.0409 with a coefficient of correlation of 0.994. This equation can be used to approximately estimate the solution mix ratio of SO_4_ to AsO_4_ required to prepare a particular solid solution composition.

**Table 2:** Chemical composition of solid digest analyses of synthesized solid solution series.

Initial solution (AsO_4_)/(SO_4_+AsO_4_)	Measured solid digest concentrations in mol/L				Molar ratios Ca:Al:(SO_4_+AsO_4_) normalized to 6Ca					Solid product (AsO_4_)/(SO_4_+AsO_4_)	Final solutions in precipitation experiments		
X_initial solution_	Ca	Al	SO_4_	AsO_4_	Ca	Al	SO_4_	AsO_4_	SO_4_+AsO_4_	X_solid_	pH	log{HAsO4}	X_HAsO4, aq_
0.00	2.35	0.75	1.14	0.00	6.0	1.9	2.9	0.0	2.9	0	11.53	0	0
0.03	2.35	0.74	1.09	0.05	6.0	1.9	2.8	0.1	2.9	0.04	11.53	-5.17	0.0018
0.07	2.31	0.71	1.03	0.11	6.0	1.8	2.7	0.3	2.9	0.09	11.52	-5.12	0.0051
0.10	2.31	0.71	0.99	0.14	6.0	1.8	2.6	0.4	2.9	0.12	11.51	-4.76	0.0053
0.30	2.33	0.65	0.79	0.34	6.0	1.7	2.0	0.9	2.9	0.3	11.43	-4.82	0.0051
0.60	2.08	0.63	0.49	0.55	6.0	1.8	1.4	1.6	3.0	0.53	11.48	-4.76	0.0078
0.80	2.30	0.66	0.43	0.80	6.0	1.7	1.1	2.1	3.2	0.65	11.52	5.2	0.0203
1.00	2.11	0.45	0.00	1.12	6.0	1.3	0.0	3.2	3.2	1	11.54	-4.85	1

**Fig 1:**
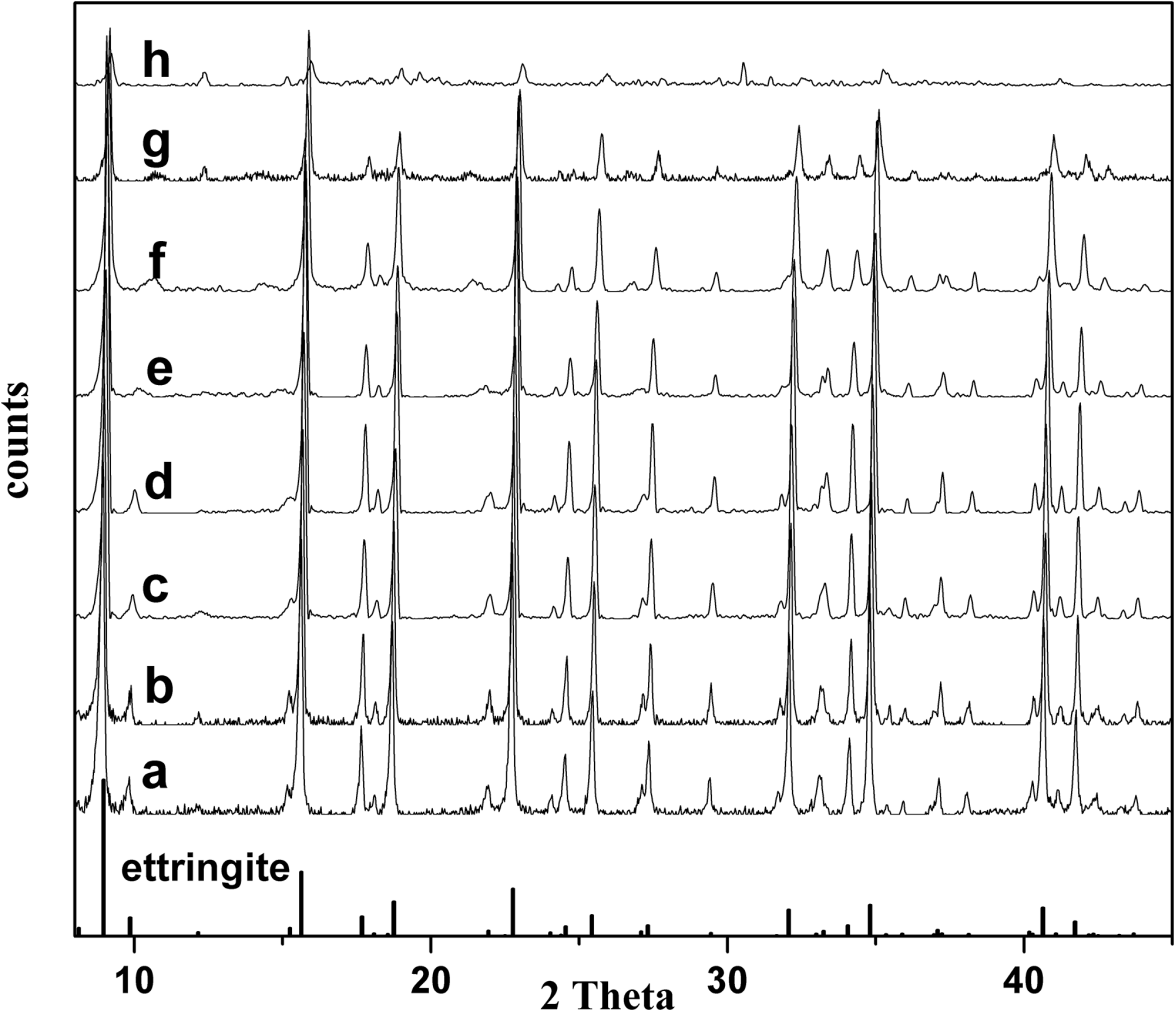
X-ray diffractograms of the solid solution series of AsO_4_–ettringite and SO_4_–ettringite. a, b, c, d, e, f, g, and h correspond to the samples with *X*_solid_ of 0, 0.04, 0.09, 0.12, 0.30, 0.53, 0.65, and 1.00, respectively.

Sharp peaks in the X-ray diffractograms (Fig 1) of the synthesis products indicated good crystallinity, and ettringite was the only stable crystalline phase except the sample h. Although the low intensities of the diffraction peaks are obtained in the pure As-sample (Fig 1h), the ettringite phase clearly can be recognized. The peaks showed slight shifts to higher angles (smaller *d*-spacing) with substitution of AsO_4_ for SO_4_ (including the sample h). This decrease in basal spacing implied that the interchannel for sulfate and water molecules was compressed after AsO_4_ uptake, which can be attributed to the intercalation of an arsenate anion in exchange for sulfate and the displacement of the ordered water molecules [26, 27]. The solids with high arsenic content (*X*_solid_ > 0.5) evidently had lower intensities of the diffraction peaks, which indicated lower crystallinity of samples. The peak intensity (around 9.9°) decreased with increasing As content in the solid solution series, and disappear when there was no sulfur. That was due to the elevated electron density in the structure, which resulted from the substitution of SO_4_ by AsO_4_. A new peak (around 12.6°) appeared when As contents in the samples were higher than 0.65. This could be attributed to CaAl_2_O_4_·10H_2_O. And there was another new peak at 30.4° in sample h, which was due to Ca_3_(AsO_4_)_2_·10H_2_O. The peak intensity around 37.3°rapidly decreased with As increasing in solid samples, which may be attributed to As substitute for partial H_2_O in the channel of ettringite. And the rapid decrease of the peak (37.3°) caused a false appearance of peak split in f, g and h.

**Fig 2:**
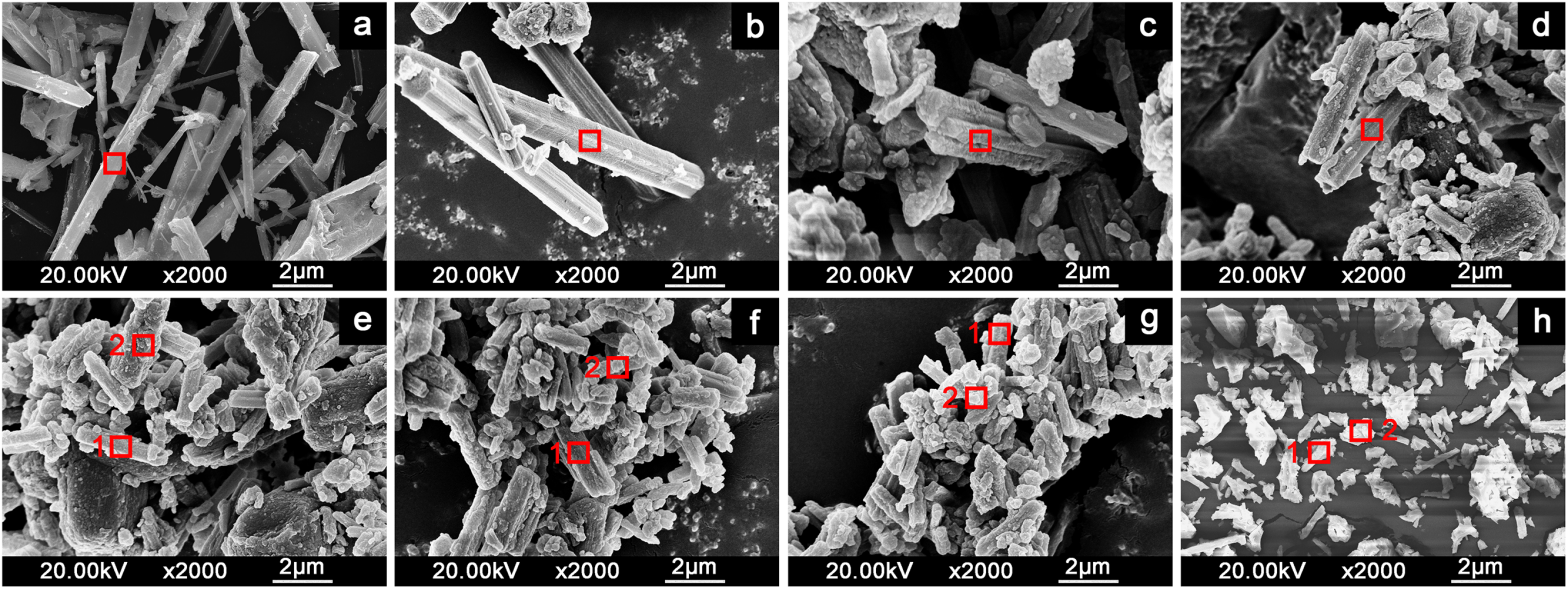
SEM micrographs of the solid solution series of AsO_4_–ettringite and SO_4_–ettringite. a, b, c, d, e, f, g, and h correspond to the samples with *X*_solid_ of 0, 0.04, 0.09, 0.12, 0.30, 0.53, 0.65, and 1.00, respectively.

**Fig 3:**
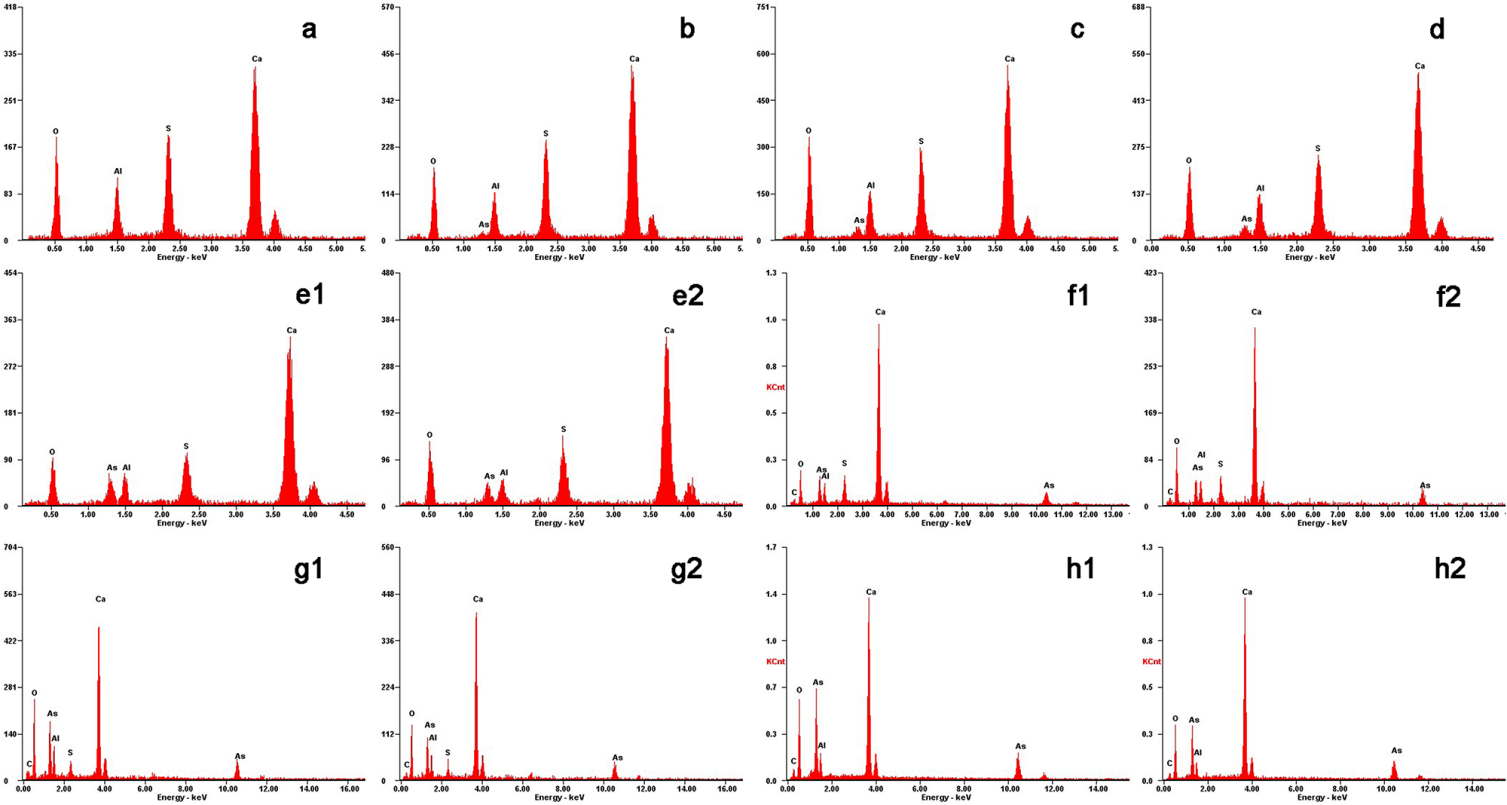
EDS mapping of the solid solution series of AsO_4_–ettringite and SO_4_–ettringite. The selected points for **EDS** were the square boxes in Fig 2.

The poorer crystalline solid with increasing As content in the samples could also be observed through the SEM micrographs of the samples (Fig 2). The club-shaped ettringite particles were approximately 5 μm to 12 μm in length when arsenic was absent. However, the grain length of ettringite decreased to <1 μm with increases in solid-phase arsenic concentration, and the length-to-diameter ratio was lower. Typical ettringite rods dominated when As contents in samples were lown(<0.3), while some irregular particles attached to the rods with short length at higher As content. The irregular particles also contained the same elements with the prisms, which were showed from EDS mapping (Fig 3).

**Fig 4:**
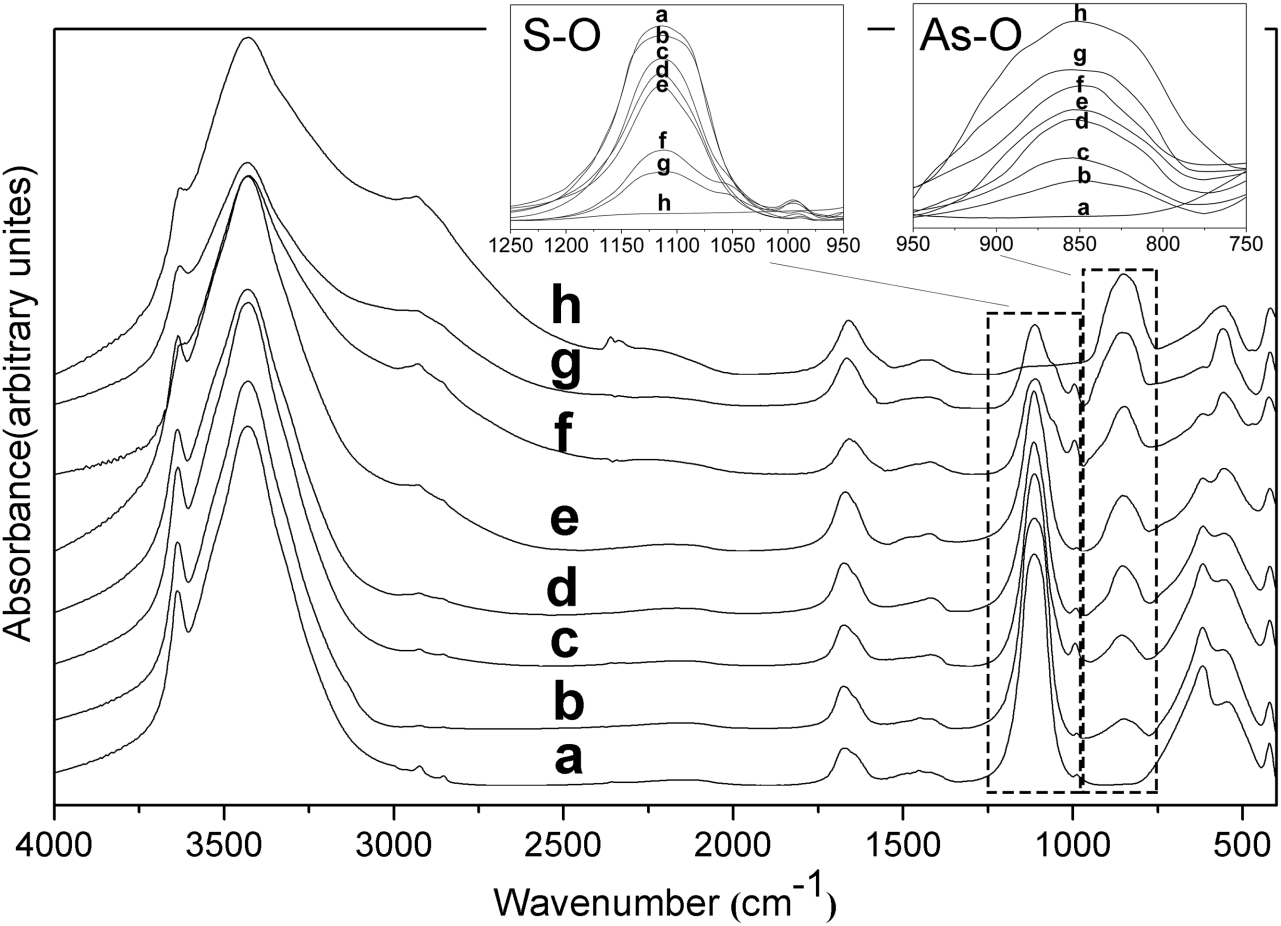
FTIR spectra of the solid solution series of AsO_4_–ettringite and SO_4_–ettringite. a, b, c, d, e, f, g, and h correspond to the samples with *X*_solid_ of 0, 0.04, 0.09, 0.12, 0.30, 0.53, 0.65, and 1.00, respectively.

Oxyanion speciation could be confirmed by Fourier transform infrared (FTIR) spectroscopy. Fig 4 shows the spectra of the solid solution series of AsO_4_-SO_4_-ettringite. The assignments of vibration are shown in Table 3. As indicated by the peak intensities, the SO_4_ concentration in the solid solutions decreased with the increase in the solid phase AsO_4_ concentration. The FTIR spectra of solid solutions exhibited an As–O stretching peak at 855 cm^−1^, which was higher than 810 cm^−1^ reported by Siebert H. [28]. AsO_4_^3-^ was protonated to form HAsO_4_^2−^, which caused the As–OH symmetric stretch to shift to higher wavenumbers [29]. Thus, AsO_4_^3−^ substituted SO_4_^2−^ inside the channels in the form of HAsO_4_^2−^, which was also demonstrated in dissolution experiments because HAsO_4_^2−^ was the dominant form of arsenic in the equilibrated solutions. Strong and broad OH bands around 3432 cm^-1^ and 2950 cm^-1^ developed with increases in solid phase AsO_4_ concentration, and the new band (around 2950 cm^-1^) may have been due to the formation of H-bonds between AsO_4_ and structural OH or H_2_O. Possible weak carbonate bands at 1421 cm^−1^ existed in the spectra [30, 31], which could indicate carbonate contamination, but in less than 5%, because no compound containing carbonate was observed from the X-ray diffractograms.

**Table 3:** IR spectra of the solid solution series of AsO_4_–ettringite and SO_4_–ettringite and band assignments.

Frequency (cm^−1^)	Vibrations
553	Al(OH)_6_
619	SO_4_
855	HAsO_4_
1112	SO_4_
1421	Possible CO_3_
1668	H_2_O
3432	H-boned OH
3635	Non-H-boned OH

### Solid solution solubility products

The ion concentrations in solution of the dissolution experiments were shown in Table 4. And the Ca:Al:SO_4_:AsO_4_ ratios in solution were different from those of the solid, suggesting non-stoichiometric dissolution. This was expected, since the presence of secondary phases (Ca-As compound, gypsum and Al-hydroxide) leaded to reduction of dissolved Ca, Al, SO4 and AsO4 concentrations in solution.

**Table 4:** Ion concentrations in solution in the dissolution experiments (mmol/L).

Sample(*X*_solid_)	Ca	Al	SO_4_	AsO_4_	pH	log{HAsO4}	X_HAsO4,aq_
0.00	2.984	1.032	5.261	0.000	10.85	0	0
0.04	3.004	1.010	5.105	0.020	10.80	-5.3	0.0019
0.09	2.692	0.857	4.452	0.081	10.90	-4.89	0.0053
0.12	2.677	0.811	4.067	0.088	10.97	-4.91	0.0056
0.30	2.530	0.842	4.315	0.094	11.09	-4.94	0.0049
0.53	1.745	1.912	2.902	0.131	11.32	-4.87	0.0082
0.65	1.836	2.206	1.268	0.160	11.34	-4.86	0.0186
1.00	1.751	1.579	0.000	0.174	11.41	-5.06	1

The chemical speciation of the ions and saturation index (SI) calculations were performed using the geochemical speciation model Visual MINTEQ 3.1. Activity coefficients of aqueous species were calculated with the geochemical speciation model Visual MINTEQ 3.1 using the Davies equation:

$$\text{log}\gamma_{i}=-AZ_{i}\left(\frac{\sqrt{I}}{1+\sqrt{I}}-0.24I\right)$$ (1)

In the precipitation experiments, the results showed the saturation index (SI) of ettringite, Ca_4_(OH)_2_(AsO_4_)_2_·4H_2_O, Ca_3_(AsO_4_)_2_·xH_2_O at low molar ratio (X _initial solution_, <0.3), and ettringite, Al(OH)_3_, boehimte, Ca_5_(AsO_4_)_3_OH, Ca_3_(AsO_4_)_2_·xH_2_O, biaspore, gibbsite at higher molar ratio, and Al(OH)_3_, boehimte, Ca_5_(AsO_4_)_3_OH, Ca_3_(AsO_4_)_2_·xH_2_O, diaspore, gibbsite, without sulfate were above 0. However, ettringite was the only the stable phase except the sample without sulfate. This indicated the content of the other phases was below XRD detection limit even though these phases were present. However, both the XRD result of the sample without sulfate and the saturation index (SI) indicated Ca_3_(AsO_4_)_2_·10H_2_O was present. In the dissolution experiments, the saturation index (SI) of Al(OH)_3_, boehimte, Ca_5_(AsO_4_)_3_OH, diaspore, ettringite, gibbsite at X _solid_ <0.3, and Al(OH)_3_, Boehimte, Ca_5_(AsO_4_)OH, diaspore, ettringite, gibbsite, Ca_4_(OH)_2_(AsO_4_)_2_·4H_2_O at higher X _solid_ were above 0.

FTIR spectroscopy analysis of the solid samples and chemical analysis of the liquid phase revealed that AsO_4_^3−^ substituted for SO_4_^2−^ inside the channels in the form of HAsO_4_^2−^. Thus, HAsO_4_^2−^ will be used for the thermodynamic study of the solid solution series.

The solubility products of the solid solution series was calculated according to the following reaction:

$$\text{log}K_{\text{sp}}=6\text{ log}\left\{{\text{Ca}}^{2+}\right\}+2\text{ log}\left\{\text{Al}{\left(\text{OH}\right)}_{4}{}^{-}\right\}+3\left[x\text{log}\left\{{\text{HAsO}}_{4}{}^{2-}\right\}+\left(1-x\right)\text{log}\left\{{\text{SO}}_{4}{}^{2-}\right\}\right]+4\text{ log}\left\{{\text{OH}}^{-}\right\}+26{\text{log}\{\text{H}}_{2}\text{O}\}$$ (2)

**Fig 5:**
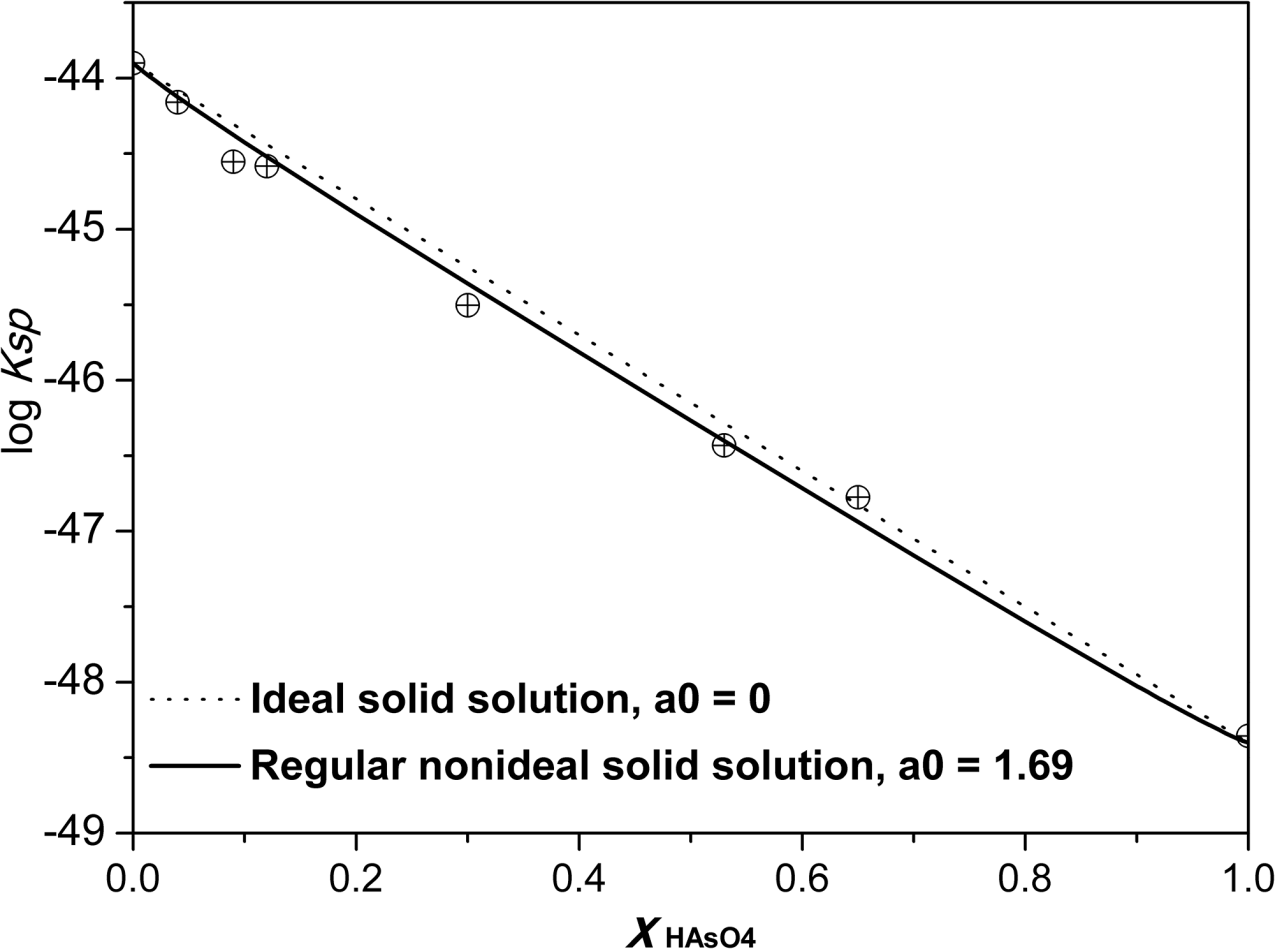
Solid mole fraction/solubility products plot for the system HAsO_4_–ettringite and SO_4_–ettringite solid solution at 25°C. The calculated *Ksp* of the solid solutions fits best to the nonideal model.

The curly brackets {} denote aqueous activities. The *Ksp* calculated are shown in Fig 5. The calculated AsO_4_–ettringite and SO_4_–ettringite solubility products change as a function of *X*_HAsO4_ for mixed phases, with a linear correlation between log*Ksp* and *X*_HAsO4_. This finding indicates that solid solutions exist. Solubility calculations of all precipitation and dissolution experiments resulted in a mean log*K*_As-ettringite_ = -48.4 ± 0.4 and *K*_SO4–ettringite_ = -43.9 ± 0.6. The solubility of SO_4_–ettringite in the current study was higher than that determined by Barbara and Thomas (-44.9) [14, 32], which could be due to a small CO_2_ amount in the system that lowers the pH. Certainly other factors, such as the choice of the activity coefficient model, analytical errors, and the presence of other complexes not included in the activity calculations, may affect estimates based on minimizing the variance in *Ksp*.

The aqueous solubility of the binary solid solution system HAsO_4_–ettringite and SO_4_–ettringite could be predicted by plotting the Lippmann’s solidus and solutus relationships on the ordinate against two superimposed scales, i.e., *X*_HAsO4_ and *X*_HAsO4,aq_, on the abscissa, which could provide the solid-phase and aqueous-phase compositions for a series of possible thermodynamic equilibrium states [33–35]. In this case, the solidus and solutus equations can be expressed as follows:

$$\begin{array}{ll} {\Sigma \Pi }_{\text{eq}} & ={\left\{{\text{Ca}}^{2+}\right\}}^{6}{\left\{\text{Al}{\left(\text{OH}\right)}_{4}{}^{-}\right\}}^{2}{\left[\left\{{\text{SO}}_{4}{}^{2-}\right\}+\left\{{\text{HAsO}}_{4}{}^{2-}\right\}\right]}^{3}{\left\{{\text{OH}}^{-}\right\}}^{4}{\left\{{\text{H}}_{2}\text{O}\right\}}^{26}\\ & =K_{{\text{SO}}_{4}}X_{{\text{SO}}_{4}}\gamma_{{\text{SO}}_{4}}+K_{{\text{HAsO}}_{4}}X_{{\text{HAsO}}_{4}}\gamma_{{\text{HAsO}}_{4}} \end{array}$$ (3)

and

$${\Sigma \Pi }_{\text{eq}}=\frac{1}{\frac{X_{{\text{HAsO}}_{4,\text{aq}}}}{K_{{\text{HAsO}}_{4}}\gamma_{{\text{HAsO}}_{4}}}+\frac{X_{{\text{SO}}_{4,\text{aq}}}}{K_{{\text{SO}}_{4}}\gamma_{{\text{SO}}_{4}}}}$$ (4)

where the curly brackets {} denote aqueous activities; *X*_HAsO4_ and *X*_SO4_ are the mole fractions of HAsO_4_ and SO_4_ (*X*_SO4_ + *X*_HAsO4_ = 1) in the solid, respectively; *X*_HAsO4,aq_ and *X*_SO4,aq_ are the activity fractions of HAsO_4_^2−^ and SO_4_^2−^ ions in the aqueous solution, respectively; *K*_HAsO4_ and *K*_SO4_ are the solubility products of pure HAsO_4_–ettringite and SO_4_–ettringite, respectively; and *γ*_*HA*sO4_ and *γ*_SO4_ are solid activity coefficients.

The solid-phase activity coefficients determined from the modified Guggenheim regular excess free energy model [36]can be expressed as follows:

$$\text{ln}\gamma_{{\text{HAsO}}_{4}}=X_{{\text{SO}}_{4}}{}^{2}{[\text{a}}_{0}{-\text{a}}_{1}\left(3X_{{\text{HAsO}}_{4}}-X_{{\text{SO}}_{4}}\right){+\text{a}}_{2}\left(X_{{\text{HAsO}}_{4}}-X_{{\text{SO}}_{4}}\right)\left(5X_{{\text{HAsO}}_{4}}-X_{{\text{SO}}_{4}}\right)+\ldots ]$$ (5)

$$\text{ln}\gamma_{{\text{SO}}_{4}}=X_{{\text{HAsO}}_{4}}{}^{2}{[\text{a}}_{0}{-\text{a}}_{1}\left(3X_{{\text{SO}}_{4}}-X_{{\text{HAsO}}_{4}}\right){+\text{a}}_{2}\left(X_{{\text{SO}}_{4}}-X_{{\text{HAsO}}_{4}}\right)\left(5X_{{\text{SO}}_{4}}-X_{{\text{HAsO}}_{4}}\right)+\ldots ]$$ (6)

Regular nonideal solid solutions only need one Guggenheim fitting parameter (*a*_0_), which was determined by the MBSSAS code [33, 37]. A value of *a*_0_ = 1.69 was obtained. Thus, Eqs 5 and 6 can be simplified as follows:

$$\text{ln}\gamma_{{\text{HAsO}}_{4}}=X_{{\text{SO}}_{4}}{}^{2}{\text{a}}_{0}$$ (7)

$$\text{ln}\gamma_{{\text{SO}}_{4}}=X_{{\text{HAsO}}_{4}}{}^{2}{\text{a}}_{0}$$ (8)

**Fig 6:**
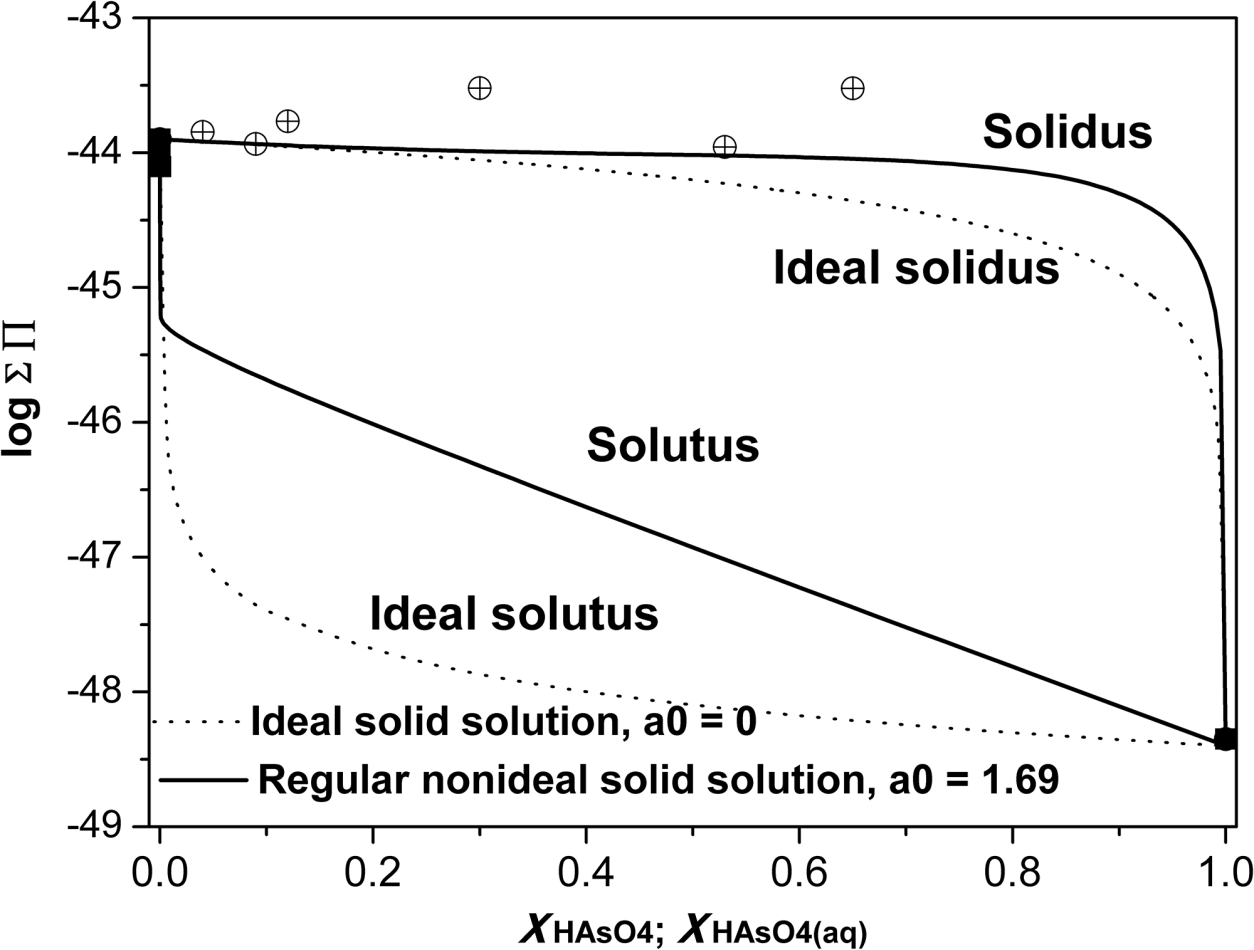
Lippmann diagram for the system HAsO_4_/SO_4_–ettringite solid solution series at 25°C.

The Lippmann diagram for this system at 25°C is shown in Fig 6. The calculated ΣΠ of the solid solution series fits best to the nonideal model.

**Fig 7:**
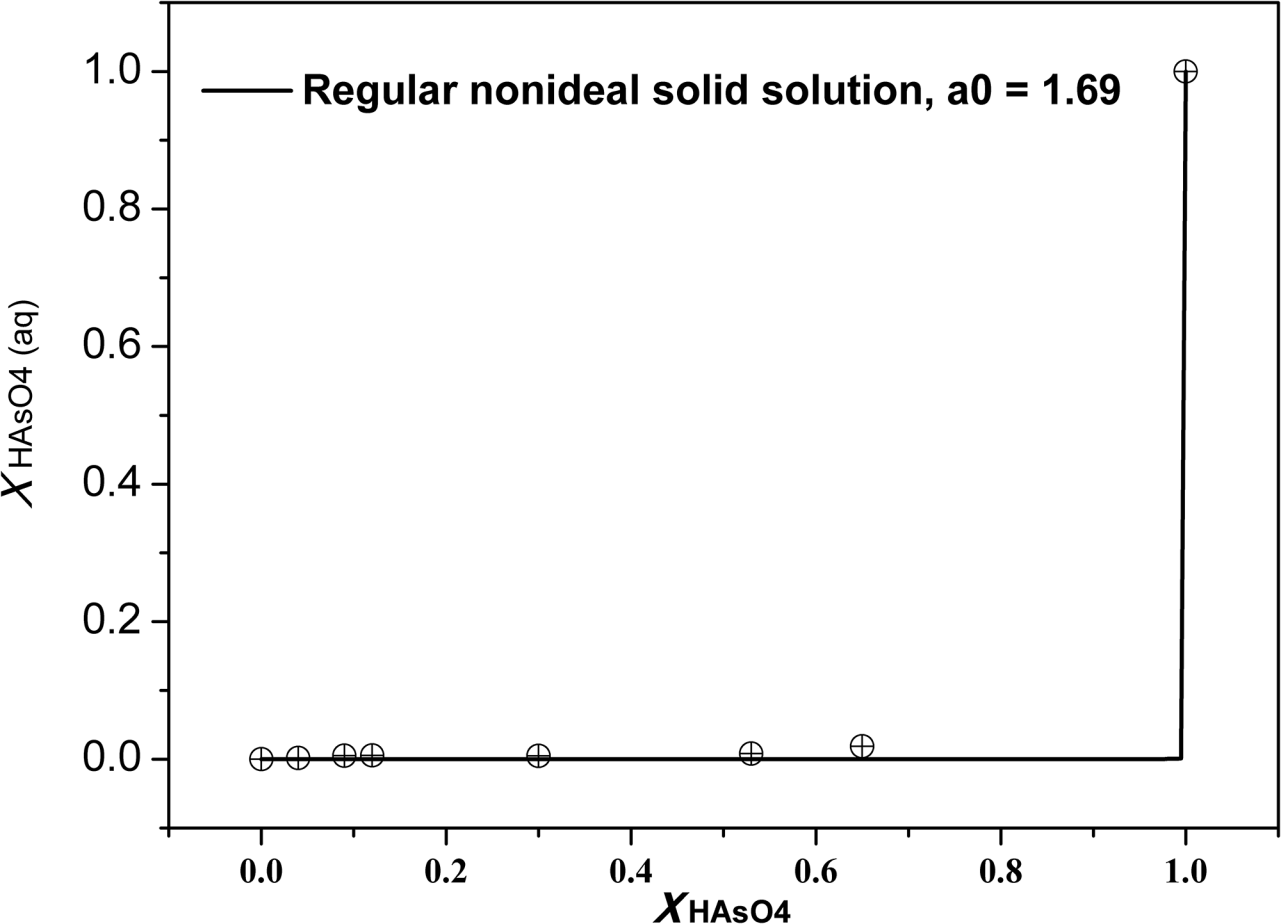
Solid mole fraction/aqueous activity fraction plot for the system HAsO_4_/SO_4_ –ettringite solid solutions at 25°C.

The pure HAsO_4_–ettringite and SO_4_–ettringite endmember solubility products in this system differ by four orders of magnitude. As a result, a strong preferential distribution of the less soluble endmember toward the solid phase was observed. This expression (Eq 9) can be used to construct a *X*_HAsO4_–*X*_HAsO4,aq_ plot (Fig 7), which describes the coexisting compositions of solid and aqueous solutions under equilibrium conditions.

$${\text{X}}_{{\text{HAsO}}_{4}}=\frac{K_{{\text{SO}}_{4}}X_{{\text{SO}}_{4}}\gamma_{{\text{HAsO}}_{4,\text{aq}}}}{\left(K_{{\text{SO}}_{4}}\gamma_{{\text{SO}}_{4}}-K_{{\text{HAsO}}_{4}}\gamma_{{\text{HAsO}}_{4}}\right)X_{{\text{HAsO}}_{4,\text{aq}}}+K_{\text{SO}4}\gamma_{{\text{SO}}_{4}}}$$ (9)

Fig 7 shows that the *X*_HAsO4_–*X*_HAsO4,aq_ curve approximates to two straight lines forming a right angle, which implies that HAsO_4_-poor aqueous solutions are in equilibrium with HAsO_4_-rich solid phases in a wide *X*_HAsO4_ range.

In summary, ettringite containing SO_4_^2−^ or HAsO_4_^2−^ has been synthesized, and its thermodynamic data were first published. The results revealed that HAsO_4_–ettringite and SO_4_–ettringite solid solutions exist and that this solid solution had HAsO_4_-poor aqueous solutions in equilibrium. This finding implies that ettringite (AFt-phase) may play an important role in AsO_4_^3−^ solidifying mechanisms in the cement matrix and should be considered in AsO_4_^3−^ leaching modeling projects.

## Supporting information

S1 File X-ray diffraction data of the solid solution series of AsO4- and SO4-ettringites. a, b, c, d, e, f, g, and h correspond to the samples with Xsolid of 0, 0.04, 0.09, 0.12, 0.30, 0.53, 0.65, and 1.00, respectively.
(ZIP)

S2 File FTIR spectral data of the solid solution series of AsO4- and SO4-ettringites. a, b, c, d, e, f, g, and h correspond to the samples with *X*_solid_ of 0, 0.04, 0.09, 0.12, 0.30, 0.53, 0.65, and 1.00, respectively.
(ZIP)
